# Evaluation of Awareness, Knowledge, Attitude and Practices Related to Toxoplasmosis Among Females in Algeria

**DOI:** 10.3390/vetsci12010010

**Published:** 2024-12-29

**Authors:** Mohamed Lounis, Samah Aissaoui, Fatima Ghouissem, Karim Souttou

**Affiliations:** 1 Department of Agro-Veterinary Sciences, Faculty of Natural and Life Sciences, University of Djelfa, PO Box 3117, Djelfa 17000, Algeria; 2 Laboratoire d’Exploration et Valorisation des Écosystèmes Steppiques, Faculty of Natural and Life Sciences, University of Djelfa, PO Box 3117, Djelfa 17000, Algeria; k.souttou@univ-djelfa.dz; 3 Department of Biology, Faculty of Natural and Life Sciences, University of Djelfa, PO Box 3117, Djelfa 17000, Algeria; aissaoui.samah2001@gmail.com (S.A.); fatimaghouissem1@gmail.com (F.G.)

**Keywords:** Algeria, attitude, awareness, knowledge, toxoplasmosis

## Abstract

Toxoplasmosis is a major veterinary and public health issue affecting humans and animals, where felids represent the definitive hosts of the causative agent *Toxoplasma gondii*. While the infections are mostly asymptomatic, the disease could be severe in immune-compromised patients and pregnant women. This study was conducted to evaluate the level of knowledge, practice, and attitude toward this disease among a sample of Algerian women. A cross-sectional poll was conducted between 25 March and 28 August 2024, using a self-administered questionnaire. Results showed that only 53% of the 545 respondents heard of the disease before this survey, and being married increased the odds of awareness (AOR: 3.597 95% CI: 1.598–8.099). Among those who heard of the disease (288 women), 52.7% correct responses were obtained with varied frequencies regarding the mode of transmission and symptoms. The participants were aware of the role of cats as a source of contamination for humans (83.3%) and the common routes of transmission, including contact with cat feces (83.7%) and the consumption of undercooked meat (75%), but the role of unpasteurized milk from infected animals (40.3%), untreated water (35.8%), and blood transfusions (26.4%) was not well understood. The majority of participants ignored symptoms such as skin rashes (29.2%) and swollen lymph nodes (36.5%), as well as sequelae including mental impairment (32.3%) and eyesight issues (35.1%). Results showed also a high rate of appropriate practices and positive attitudes, where 90.3% of the participants said they were in favor of any action that intended to control toxoplasmosis. These results suggest the need to increase the level of awareness of Algerian women regarding this disease.

## Simple Summary

Toxoplasmosis is a parasitic disease that can affect humans and animals. One of the high-risk categories of severe cases of toxoplasmosis is pregnant women. In fact, if the causative agent is transmitted to the fetus, the latter could suffer from severe neurologic and ophthalmic signs. In this way, this study aimed to determine if Algerian women of reproductive age are aware of this disease, what the level of knowledge is, and what their attitudes and practices are to prevent it. Results showed that nearly half of the 545 asked women had not heard of the disease from where a medium level of knowledge was obtained with a mean of 52.7% of correct responses. Notwithstanding these results, appropriate practices and positive attitudes were obtained. These results demonstrate the need to raise awareness and knowledge of Algerian women about the danger of this parasitic zoonosis.

## 1. Introduction

Toxoplasmosis represents a serious veterinary and public health problem worldwide [1]. While it affects about one-third of the human global population [2], it affects a wide range of warm-blooded animal species [3], including the definitive host, mainly cats and other felids, and multiple intermediate domestic and wild hosts (mammals and birds). In fact, it is reported among most farmed animals (mainly sheep, goats, cows, and pigs, and less frequently in chickens, rabbits, horses, and camels [2,4], constituting a major obstacle for livestock production worldwide with serious economic and reproductive losses related to abortion, stillbirth, and a decrease in milk production [5]. In addition, the presence of the causative agent of the disease in the ecosystem makes the disease a one health challenge [6].

This anthropozoonosis is caused by an obligate intracellular coccidian parasite, namely *Toxoplasma gondii* (*T. gondii*). This opportunistic parasite has a complex life cycle alternating between sexual stages taking place in the definitive hosts and asexual reproduction in the intermediate hosts. The intermediate host may be infected through the ingestion of sporulated oocyst (contaminated soil, foods, and water) and the ingestion of bradyzoites in undercooked or raw infected meat [4,7]. In addition, vertical transmission across the placenta from the mother to the fetus could also occur [7]. The role of contaminated milk has also been reported in recent years [8].

Clinically, even most *T. gondii* infections in humans are asymptomatic in immune-competent subjects; over one-tenth of infected individuals develop signs of lymphadenopathy, vision disorders, and mild flu-like and/or mononucleosis-like symptoms [9,10]. The signs could be more severe in immune-compromised patients and in cases of congenital infection. In immune-deficient persons, the disease could reactivate latent infections, leading to cerebral complications. In addition, the parasite could cause multiple disorders including cognitive impairment, schizophrenia, bipolar disorder, and epilepsy [11,12].

If the parasite is transmitted vertically from a pregnant woman to the fetus, the signs could include fetal death, stillbirth, malformation, abortion, central nervous system abnormalities at birth, or ocular anomalies of the child. The severity of these signs is however related to the stage of pregnancy and the effectiveness of the placental barrier [4,13,14]. In fact, the risk of vertical transmission is higher in the third trimester, but the clinical manifestations are more severe when fetal infection occurs during the first trimester of pregnancy [13,14,15]. Congenital infections are also reported among farmed animals, especially among small ruminants with nearly the same symptomatic figure (abortions, stillbirths, and other specific and nonspecific clinical manifestations) [3,5,7]. While the pathogenesis of the infection is not well elucidated among the other species (rabbit, horses, camel, poultry, etc.), the serological confirmation of the parasite was reported in multiple countries [4].

In Algeria, *T. gondii* infection is omnipresent among both humans and animals [7,16]. In the absence of national official data, the reported prevalence is around 50% among both pregnant women and blood donors [17,18]. For animals, a systematic review reported that the seroprevalence reported in Algeria was 20% in cattle, 22.6% in sheep, 33.6% in goats, 28.2% in horses, 30% in donkeys, 70.3% in stray cats, 14.6% in local rabbits, 30.5% in dogs, and 50.7% in poultry farms [19].

Thus, controlling this disease is vital to improve the quality of life and reduce deaths in humans as well as in animals [3]. In the absence of an effective vaccine, the essential step to accomplish this task is to increase awareness of the population of the facets of this disease and its prevention. Therefore, educating the population and providing them with appropriate information will directly affect their attitude and practices toward the disease, which will consequently help in its prevention [20,21]. In this way, evaluation of the level of knowledge of the population about the disease could help to define the gaps in knowledge that should be used for any awareness campaigns. Multiple studies were conducted to evaluate the level of knowledge, attitude, and practice (KAP) related to toxoplasmosis among different categories around the world [8,9,10,13,14,15,20,21,22,23,24]. To our knowledge, no such studies were conducted in Algeria. Thus, the current study aimed to evaluate the level of knowledge, attitude, and practices related to toxoplasmosis in a sample of Algerian women.

## 2. Materials and Methods

A cross-sectional online survey was carried out among women in Algeria from 25 March to 28 August 2024. Included in the survey were all women currently living in the Algerian territory, aged more than 18 years, and able to read and understand Arabic and/or French languages. Algerian women who currently live in another country or women of other nationality living in Algeria were excluded from the study. The survey used a questionnaire that was prepared in Google Forms, and the URL link was disseminated through social media platforms (mainly Facebook, Messenger, and WhatsApp).

Electronic consent was required before enrollment of the participant in the survey. Participants were assured that the analysis would be completely anonymous, and no personal information was required. This study was approved by the scientific committee of the Nature and Life Science Faculty, Djelfa University. The STROBE guidelines for cross-sectional studies were followed [25].

To estimate the minimal number of required females in the sample, an online sample size calculator [26] was used with the following assumptions: 15,000,000 women aged over 18 years old for the year 2024, 5% margin of error, 95% confidence interval. The minimum sample size was 385 women.

The questionnaire that was prepared from previous studies related to toxoplasmosis knowledge, attitude, and practice [9,10,13,14,15] comprised 28 close-ended questions that were divided into five groups including demographic characteristics, awareness of toxoplasmosis, knowledge, practice, and attitude toward toxoplasmosis prevention. The demographics included age, educational attainment, marital status, having children, and owning a cat. The awareness section contains three items asking the participants if they ever heard of the disease before this survey and how they heard of it. They were also asked if they knew one who was affected by this disease. The knowledge section contains different items related to the perception of the zoonotic character of the disease and its severity, the routes of transmission, the symptoms and complications, and the diagnosis, treatment, and prevention of the disease. The practice sections comprise eight items related to some habits and behaviors that could help in the dissemination or in the prevention of the disease. At last, the participants were asked about their implication in awareness campaigns and their attitude toward any awareness campaigns to prevent the disease.

### Statistical Analysis

Using Excel 2007 and SPSS Version 22, the statistical analysis was carried out, including both descriptive and inferential statistics. The overall responses and demographic traits of the participants were compiled using descriptive statistics that include the calculation of frequencies and percentages (%).

For inferential statistics, the Pearson chi-square test, crude Odds Ratio (COR) and adjusted OR (AOR) were used in order to find the possible relationship between the dependent and independent variables. The chi-squared test and COR were used to find the relation between the demographic characteristics and the awareness of the disease. The analysis was completed by the calculation of AOR for significant variables. COR was also used to calculate the association between awareness of the disease and the adoption of appropriate practices among the participants. A *p*-value of less than 0.05 was specifically regarded as indicating statistical significance for all analyses.

## 3. Results

### 3.1. Sociodemographics and Awareness of the Diseases

A total of 545 women filled out the questionnaire for this study. By removing two incomplete responses, the total number was reduced to 543. Almost all respondents (95%) have a university degree, and more than four-fifths (80.8%) were single. In addition, nearly two-thirds of the study population were aged between 20 and 30 years old, while just over 34% declared owning a cat.

Out of the total participants, 53% declared being aware of this disease before their enrollment in this survey, and 16.7% among them declared knowing one who was affected by toxoplasmosis (Table 1).

In the bivariate analysis, aged women of more than 40 years (82.4%), and those 31 and 40 years (80.2%) were more aware of the disease than those aged between 20 and 30 (52.5%) and less than 20 years old (34%) (*p* = 0.000). In addition, married individuals (78.8%) showed the highest level of awareness than singles (46.9%) (*p* = 0.000).

The multiple regression model shows that, compared to individuals less than 20 years old, those aged between 31 and 40 years (AOR: 3.434, 95% CI: 1.398–8.735), and 20–30 years old (AOR: 1.966, 95% CI: 1.239–3.119) showed the highest odds of awareness. Married women showed an AOR of awareness of 3.597 (95% CI: 1.598–8.099) while pregnancy was not a determinant even though it showed a significant COR of 2.963 (95% CI COR: 1.102–7.965) (Table 2).

**Figure 1:**
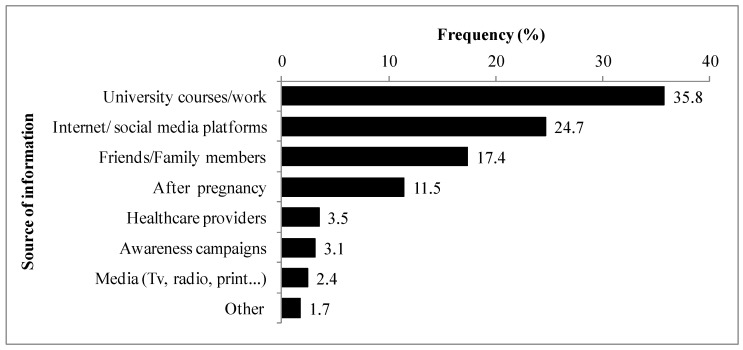
Source of information about toxoplasmosis of the study population.

When asked about how they heard of the disease, university courses and work were the main source (34.5%) followed by the Internet/social media platforms (24.7%) and friends and family discussions (17.4%), while 11.4% declared being aware of the disease after their pregnancy (Figure 1).

### 3.2. Knowledge About Toxoplasmosis

From the 288 women who heard of toxoplasmosis, 59.7% claimed that this condition is a serious disease, while 86.1% knew that this disease is zoonotic.

Regarding the source of transmission, 83.3% were aware of the role of cats as a source of contamination for humans, while 83.7% and 75% were aware that foods being in contact with cat feces and consuming undercooked meat represent the main way of infection, respectively. Low levels of awareness were obtained regarding the role of unpasteurized milk from affected animals (40.3%), untreated water (35.8%), and blood transfusion (26.4%). The participants knew that pregnant women (94.4%) and immune-compromised people (69.4%) were the persons at risk for toxoplasmosis, while only a small portion of 21.9% were aware of the sensibility of aged individuals.

Thirty-three percent (33%) of the asked women claimed that toxoplasmosis is in general asymptomatic among pregnant women, and fever and flu-like symptoms (62.8%) were the most cited symptoms, while fetal malformation (83%), miscarriage, and stillbirth (58.7%) were the most known complications of the disease.

Out of the total respondents, 83% declared that women who have been infected with *T. gondii* during pregnancy can transmit it to their baby, while 24.0% declared that they can transmit the disease to the fetus if they contract it before their pregnancy. A total of 60.8% declared that the highest period of severity of lesions for the fetus is when the disease is contracted in the first trimester.

At last, more than half of the participants (52.1%) declared that the disease can be treated in pregnant women, 70.5% were aware of the availability of diagnosis tests during pregnancy, and 22.9% were aware of the unavailability of a vaccine against this disease (Table 3).

### 3.3. Toxoplasmosis Related Practices

Results showed that for all of the asked items related to practices, the lowest rate of good practice was 50.8% (for the item “*avoiding consumption of unpasteurized milk*”) and the highest rate was 94.8% (for the item “*washing fruit and vegetables before consumption*”). In addition, except for the items “*eating undercooked meat*” (COR: 1.594, 95% CI 1.090–2.332), “*eating fresh salad without making sure it is washed well*” (COR: 1.43, 95% CI: 0.972 to 2.103), and “*avoid contact with stray cats*” (COR: 1.4295, 95% CI: 0.972–2.103), awareness of toxoplasmosis significantly increased the odds of good practices (Table 4).

### 3.4. Attitude Toward Toxoplasmosis Prevention

**Figure 2:**
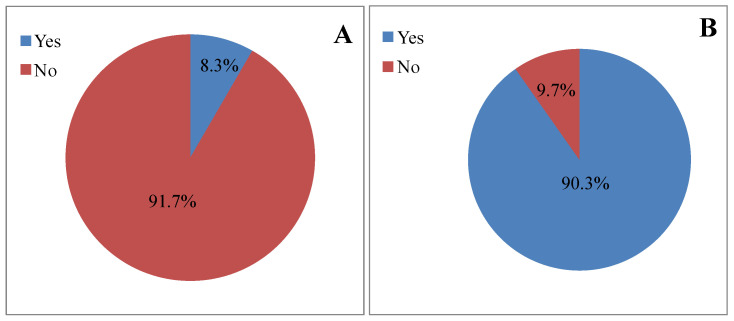
Attitude of the study population toward preventive actions against toxoplasmosis. (**A**): Have you ever participated in any training, awareness campaign or workshop related to this disease? (**B**): Do you support any initiative aimed at controlling toxoplasmosis?

Results presented in Figure 2A,B showed that 8.3% of the participants declared participating in trainings, awareness campaigns, or workshops related to toxoplasmosis, while 90.3% declared that they support any initiative aimed at controlling toxoplasmosis.

## 4. Discussion

This survey was conducted with the aim to assess awareness, knowledge, attitude, and practices related to toxoplasmosis—a disease widely present among humans and animals in the country [17,18,19]—among women in Algeria. This is the first study dealing with this subject in Algeria. Its results would help in the prevention strategies against this disease. In fact, the lack of knowledge about toxoplasmosis is considered a significant risk factor for positive seroconversion among pregnant women [27,28]. Thus, education of the population about the disease will certainly affect their attitude toward the disease, thus reducing the risk of infection [21,29].

The first apparent result of this study is the lack of awareness of toxoplasmosis among the study population. In fact, 47% of the asked women declared that they had not heard of the disease before this survey. This percentage is lower than the reported levels of awareness in Saudi Arabia (79%) [23] and Italy (84%) [30], but higher than the level reported in Nigeria (42%) [31] and Palestine (38.25%) [15]. This result could be explained by the fact that most the participants were young females of less than 30 years. In fact, the level of awareness increased significantly with age. The same result was also reported in the neighboring country of Morocco in young individuals like university students (47.2%) [13], while a higher level (79.5%) was reported among medical students in Libya [32]. In the same way, married women were more than three times more aware of the disease than singles in our study (OR: 3.597 (95% CI: 1.598–8.099), while pregnancy has not shown an effect on the level of awareness. Of note, low levels of awareness were also reported among pregnant women in Morocco [9,33] and in Turkey [8]. Thus, education of pregnant women about toxoplasmosis is of great importance to improve their knowledge, attitudes, and the ability to recognize early signs of toxoplasmosis and prevent its complications [34].

The second observation is that 52.7% of correct responses were obtained among women who heard of this disease. In addition, 83.3% of the participants were knowledgeable of the definitive host, and 83.7% and 75% knew that *T. gondii* infection could be acquired through contaminated foods and undercooked meat, respectively. In fact, the role of raw meat, vegetables, and shellfish has been widely documented [35,36], while the link between Toxoplasma infection and cat ownership was controversial [8,28,37,38], suggesting that the infection is mainly favored by other practices, including gardening, contact with stray cats, and/or the non-adherence to the preventive measures (hand washing or wearing gloves) following a contact with animals [28]. These levels are higher than those reported among different categories in other countries, including Morocco [9,13,33], Egypt [29], Saudi Arabia [24], Turkey [8], and Palestine [15]. The lack of knowledge of our participants is, however, related to the role of untreated water (40.3%) and blood transfusion (26.4%). These levels are lower than those reported from pregnant women in Morocco [9] and female students in Egypt [29]. A low proportion was also reported among healthcare workers in Morocco regarding the role of untreated water (30.1%) [14]. These results could be explained by the fact that the implication of drinking water as a potential source of contamination dates only to recent years [39]. Since then, multiple water-bone outbreaks of toxoplasmosis were reported in different countries [9,13]. In addition, the consumption of unboiled shallow well water was shown to be positively correlated with *T. gondii* antibodies, especially among poorly hygienic farms [40]. Unsafe water consumption was also significantly associated with *T. gondii* seroprevalence among pregnant women in Ethiopia [28]. A recent study in Algeria provided a questionable observation, reporting that the use of bottled water in summer was significantly associated with a positive seroprevalence of *T. gondii* [41]. Of note, oocysts can remain viable for long periods in water and can resist freezing and moderately high water temperatures [42]. The results of our study suggest the need to increase awareness about the water-borne character of this disease.

More importantly, nearly all participants (94.4%) were aware that pregnant women are among the high-risk group, and 83% stated that pregnant women could transmit the parasite to their fetus if the infection is contracted during their pregnancy. These points are a common observation in previous studies [9,13,43]. These results are not shocking knowing the association pregnant women–cats–toxoplasmosis is commonly known, especially if we know that university courses and Internet/social media tools were the main sources of information for the asked women.

Contrarily, low levels of correct responses were obtained for the symptoms and complications items. In fact, even fetal malformations (83%) were widely cited as a common complication and fever and influenza-like symptoms as a common sign (62.8%); the other signs like enlarged lymph nodes (36.5%) and skin rashes (29.2%) and complications like vision problems (35.1%) and mental retardation (32.3%) were ignored by most of the participants. In addition, two-thirds of participants ignored that toxoplasmosis is generally asymptomatic among pregnant women. These observations are common in previous studies showing a low level of knowledge regarding symptoms [9,13,21,29,33]. These findings are worrying knowing the importance of recognition of symptoms in the early diagnosis of the disease and thus reducing the risk of its transmission to the fetus. In addition, the knowledge about toxoplasmosis complications for the woman and her fetus may certainly lead to adopting positive preventive behaviors toward the disease [30].

The positive fact is, however, related to the fact that 70.5% of the asked women were knowledgeable of the availability of diagnosis tests for toxoplasmosis and more than half were aware of the possibility of treatment of pregnant women. Lower percentages were obtained among pregnant women in Morocco [9].

Another observation of this study is related to the practices related to toxoplasmosis. In fact, the results reported appropriate practices in most of the items, with rates varying from 50.8% (avoiding drinking raw milk) to 94.8% (washing fruit and vegetables before consumption). More interestingly, almost all appropriate practices were significantly associated with the awareness of toxoplasmosis. These results are in line with the findings of Aldali et al. in Saudi Arabia [23] and those reported among pregnant women in Morocco [9], but higher than in Armenia-Quindío (Colombia, South America) [21] and among female students in Egypt [29]. These results are encouraging, knowing the importance of these practices in the transmission of the disease. In fact, a study in Turkey found that cat ownership was associated with more than 10 times the risk of contracting toxoplasmosis [8]. In addition, a study among females in Riyadh (Saudi Arabia) found that participants who often ate medium-cooked meat were more likely to develop toxoplasmosis than those who did not [23], while Mwambe et al. [44] found that employed pregnant women showed a higher risk of infection. The authors suggested that these results may be due to the overconsumption of meat and probably to the consumption of raw vegetables without knowing whether they were properly washed.

There are several limitations to this research assessing Algerian women’s toxoplasmosis knowledge, attitudes, and preventive practices. The study’s cross-sectional design restricts the capacity to establish causal conclusions. In addition, response biases may be introduced due to the self-reported nature of the questionnaire. The convenient sampling method through an online survey could also introduce multiple sampling biases related to the respondents’ characteristics, access to the internet, and other factors that could affect the generalization of the results to all Algerian women.

## 5. Conclusions

In conclusion, this study provides an insight regarding knowledge, attitude, and practice regarding toxoplasmosis in Algeria. Even though a low level of awareness and knowledge were obtained among the study participants, most of the preventive practices were appropriate and were significantly associated with the awareness of the disease. In addition, almost all the asked women have shown a positive attitude in regard to supporting any initiative aimed at preventing this disease. The results of this study suggest the need for sensitizing Algerian women about the disease and the need to provide them with accurate information related especially to the symptoms and complications and all the possible routes of transmission. Conducting other surveys targeting other categories such as pregnant women in areas with less access to the Internet could also be another insight about the real level of knowledge about this disease.

## Figures and Tables

**Table 1:** Demographic characteristics of the study population.

Variables	Categories	Number	Frequency (%)
Age	Over 40 years	17	3.1
	31–40 years	61	11.2
	20–30 years	362	66.7
	Less than 20 years	103	19.0
Educational level	Secondary	27	5.0
	University	516	95.0
Marital status	Married (widowed, divorced)	104	19.2
	Single	439	80.8
Having children	Yes	76	73.1
	No	28	26.9
Cat ownership	Yes	187	34.4
	No	356	65.6
Heard of toxoplasmosis	Yes	288	53.0
	No	255	47.0
Knowing one with toxoplasmosis	Yes	48	16.7
	No	240	83.3

**Table 2:** Factors affecting awareness of toxoplasmosis of the study population.

Variable	Outcome	Heard of Toxoplasmosis		*p* Value	COR (95% CI)	*p* Value	AOR (95% CI)	*p* Value
		Yes, (n (%))	No, (n (%))					
Age	Over 40 years	14 (82.4)	3 (17.6)	**0.014**	9.067 (2.442–33.668)	**0.001**	3.277 (0.757–14.185)	0.112
	31–40 years	49 (80.3)	12 (19.7)	**<0.001**	7.933 (3.742–16.821)	**<0.001**	3.434 (1.398–8.435)	**0.007**
	20–30 years	190 (52.5)	172 (47.5)	0.715	2.146 (1.359–3.389)	**0.0011**	1.966 (1.239–3.119)	**0.004**
	Less than 20 years	35 (34.0)	68 (66.0)	**<0.001**	.	.	.	.
Educational level	Secondary	12 (44.4)	15 (55.6)	0.359	0.696 (0.319–1.515)	0.361	.	.
	University	276 (53.5)	240 (46.5)	.	.	.	.	.
Marital status	Married	206 (46.9)	233 (53.1)	**<0.001**	4.216 (2.54–6.996)	**<0.001**	3.597 (1.598–8.099)	**0.002**
	Single	82 (78.8)	22 (21.2)	.	.	.	.	.
Having children	Yes	64 (84.2)	12 (15.8)	**0.053**	2.963(1.102–7.965)	**0.031**	2.299 (0.801–6.579)	0.121
	No	18 (64.3)	10 (35.7)	.			.	.
Cat ownership	Yes	102 (54.5)	85 (45.5)	0.61	1.097 (0.769–1.564)	0.610	.	.
	No	186 (52.2)	170 (47.8)	.	.	.	.	.

Bold characters indicate significant results.

**Table 3:** Knowledge about toxoplasmosis of the study population.

	Item	Response	n	%	Total
Perception	Do you think that toxoplasmosis is a serious disease?	Yes	172	**59.7**	48.6
		No/I don’t know	116	40.3	
	Do you think that toxoplasmosis is a zoonosis?	Yes	248	**86.1**	
		No/I don’t know	40	13.9	
Toxoplasmosis can be transmitted through:	Main source	Cat	240	**83.3**	57.4
		Cattle, sheep, goat	14	4.9	
		Dog	7	2.4	
		Poultry	1	0.3	
		I don’t know	26	9.0	
	Undercooked meat	Yes	216	**75.0**	
		No/I don’t know	72	25.0	
	Drinking unpasteurized milk	Yes	116	**40.3**	
		No	172	59.7	
	Foods being in contact with cat stool	Yes	241	**83.7**	
		No/I don’t know	47	16.3	
	Drinking untreated water	Yes	103	**35.8**	
		No/I don’t know	185	64.2	
	Blood transfusion	Yes	76	**26.4**	
		No/I don’t know	212	73.6	
Persons at risk	Immuno-compromized people	Yes	200	**69.4**	61.9
		No/I don’t know	88	30.6	
	Pregnant women	Yes	272	**94.4**	
		No/I don’t know	16	5.6	
	Aged persons	Yes	63	**21.9**	
		No/I don’t know	225	78.1	
Symptoms	Can be asymptomatic among pregnant women	Yes in general	95	**33.0**	40.4
		Yes sometimes	107	37.2	
		No/I don’t know	86	29.9	
	Fever and influenza-like symptoms	Yes	181	**62.8**	
		No/I don’t know	107	37.2	
	Enlarged lymph nodes	Yes	105	**36.5**	
		No/I don’t know	183	63.5	
	Skin Rash	Yes	84	**29.2**	
		No/I don’t know	204	70.8	
Complications	Misscariage or stilbirh (complication)	Yes	169	**58.7**	52.3
		No/I don’t know	119	41.3	
	Fetal malformations	Yes	239	**83.0**	
		No/I don’t know	49	17.0	
	Visions problems of the child	Yes	101	**35.1**	
		No/I don’t know	187	64.9	
	Mental retardation of the child	Yes	93	**32.3**	
		No/I don’t know	195	67.7	
Toxoplasmosis and pregnancy	Can women who are infected with *T. gondii* during pregnancy transmit it to the baby?	Yes	239	**83.0**	59.7
		No	10	3.5	
		I don’t know	39	13.5	
	Can women infected with *T. gondii* before they get pregnant transmit it to the baby?	Yes	69	24.0	
		No	102	**35.4**	
		I don’t know	117	40.6	
	The highest period of severity of lesions for the fetus during	The first trimester	175	**60.8**	
		The second trimester	69	24.0	
		The third trimester	44	15.3	
Treatment and prevention	Can toxoplasmosis be treated in pregnant women?	Yes	150	**52.1**	48.5
		No/I don’t know	138	47.9	
	Are you aware of the availability of diagnosis tests of the disease?	Yes	203	**70.5**	
		No/I don’t know	85	29.5	
	Is a human vaccine against toxoplasmosis available?	No	66	**22.9**	
		Yes	76	26.4	
		I don’t know	146	50.7	
Total					52.7

Bold characters indicate correct responses.

**Table 4:** Toxoplasmosis-related practices of the study population.

Item	Response	Total		Heard of Toxoplasmosis			
				Yes		No	
		n	%	n	%	n	%
Washing hands after handling meat	Yes	491	90.4	269	54.8	222	45.2
	No	52	9.6	19	36.5	33	63.5
	**COR (95% CI): 2.105 (1.165–3.803), *p* = 0.014**						
Washing hands after contact with a cat	Yes	379	69.8	215	56.7	164	43.3
	No	164	30.2	73	44.5	91	55.5
	**COR (95% CI): 1.634 (1.130–2.364), *p* = 0.009**						
Avoiding drinking raw milk	Yes	276	50.8	162	58.7	114	41.3
	No	267	49.2	126	47.2	141	52.8
	**COR (95% CI): 1.590 (1.133–2.233), *p* = 0.007**						
Avoiding drinking untreated water	Yes	395	72.7	222	56.2	173	43.8
	No	148	27.3	66	44.6	82	55.4
	**COR (95% CI): 1.594 (1.090–2.332), *p* = 0.016**						
Eating undercooked meat	No	481	88.6	260	54.1	221	45.9
	Yes	62	11.4	28	45.2	34	54.8
	COR (95% CI): 1.429 (0.840–2.430), *p* = 0.188						
Washing fruit and vegetable before consumption	Yes	515	94.8	283	55.0	232	45.0
	No	28	5.2	5	17.9	23	82.1
	**COR (95% CI): 6.099 (2.299–16.182), *p* = 0.009**						
Eating fresh salad without making sure it is washed well	No	497	91.5	266	53.5	231	46.5
	Yes	46	8.5	22	47.8	24	52.2
	COR (95% CI): 1.256 (0.686- 2.299), *p* = 0.739						
Avoiding contact with stray cats	Yes	403	74.2	223	55.3	180	44.7
	No	140	25.8	65	46.4	75	53.6
	COR (95% CI): 1.43 (0.972–2.103), *p* = 0.0695						

Bold character indicates significant results.

## Data Availability

All of the study’s supporting data can be obtained upon request from the corresponding author.
